# Enhanced Electromagnetic Ultrasonic Thickness Measurement with Adaptive Denoising and BVAR Spectral Extrapolation

**DOI:** 10.3390/s26010216

**Published:** 2025-12-29

**Authors:** Lijun Ma, Xiaoqiang Guo, Shijian Zhou, Xiongbing Li, Xueming Ouyang

**Affiliations:** 1 Hunan Industrial Equipment Installation Co., Ltd., Changsha 410007, China; malijun@jt.hncig.cn (L.M.); guoxiaoqiang@hnaz.com.cn (X.G.); 2 School of Traffic and Transportation Engineering, Central South University, Changsha 410075, China; shijian_zhou@csu.edu.cn (S.Z.); lixb213@csu.edu.cn (X.L.)

**Keywords:** electromagnetic ultrasonic, thickness measurement, adaptive denoising, bayesian vector autoregressive

## Abstract

Electromagnetic ultrasonic testing technology, owing to its couplant-free, high-temperature-resistant, and non-contact characteristics, exhibits unique advantages for thickness measurement in harsh industrial environments. However, its accuracy is fundamentally limited by inherent constraints in signal bandwidth and low signal-to-noise ratio. To address these challenges, this work proposes an electromagnetic ultrasonic thickness measurement method that integrates Adaptive Denoising with Bayesian Vector Autoregressive (AD-BVAR) spectral extrapolation. The approach employs Particle Swarm Optimization (PSO) and automatically determines the optimal parameters for Variational Mode Decomposition (VMD), followed by integration with Singular Value Decomposition (SVD) to achieve the adaptive denoising of signals. Subsequently, the BVAR model incorporating prior constraints performs robust extrapolation of the effective frequency band spectrum, ultimately achieving high measurement accuracy signal reconstruction. The experimental results demonstrate that on step blocks with thicknesses of 3 mm and 12.5 mm, the proposed method achieved significantly reduced error rates of 0.267% and 0.240%, respectively. This performance markedly surpasses that of the conventional Autoregressive (AR) method, which yielded errors of 0.767% and 0.560% under identical conditions, while maintaining stable performance across different thicknesses.

## 1. Introduction

Electromagnetic Acoustic Transducer (EMAT) thickness measurement technology has gained significant attention in industrial non-destructive testing due to its non-contact operation and couplant-free characteristics [1,2]. These advantages make it particularly valuable for inspections in high-temperature, high-radiation, and other harsh environments [3,4]. However, the low efficiency of EMAT results in weak ultrasonic signals that are susceptible to a material’s electromagnetic properties, surface conditions, and sensor configuration [5,6,7], thereby limiting measurement accuracy.

To address these challenges, current research has evolved along two main directions. The first focuses on time-resolution enhancement through pulse compression techniques that employ coded excitation to improve signal energy. For instance, Liao et al. [8] utilized Barker codes to enhance the signal-to-noise ratio (SNR) and resolution in multi-element EMAT systems. Wang et al. [9] achieved improved defect detection capability through spatiotemporal joint optimization. Sheng et al. [10] applied Barker coding to maintain sufficient emission energy while reducing instantaneous power requirements. Nevertheless, these methods generally require complex excitation signals and high transmission power, imposing substantial demands on hardware systems. The second direction emphasizes signal processing and inversion algorithm optimization. Han et al. [11] employed time-frequency analysis to improve the interpretation of overlapping echoes, while Zhu et al. [12] combined adaptive Wiener filtering with an improved Hilbert–Huang transform to achieve high-precision extraction and the reconstruction of weak signals in noisy environments.

In contrast to the above approaches, Autoregressive (AR) spectral extrapolation has attracted growing interest in industrial non-destructive testing due to its unique capability to simultaneously compress temporal waveforms and extend frequency spectra. Researchers have demonstrated its effectiveness across various applications. Lei et al. [13] combined AR with pre-modulated linear frequency modulation excitation to achieve echo deconvolution in the high-frequency ultrasonic testing of flip-chip packages. Zhai et al. [14] employed an improved covariance-based AR model to reconstruct spectra and expand bandwidth in terahertz time-domain reflection signals. Jin et al. [15] developed a time-of-flight diffraction blind zone suppression method using AR spectral extrapolation, successfully separating lateral waves from defect tip diffractions. However, AR techniques exhibit significant sensitivity to noise in practical applications, where spectral interference substantially degrades extrapolation accuracy, necessitating effective pre-processing denoising. However, AR techniques exhibit significant sensitivity to noise in practical applications, where spectral interference substantially degrades extrapolation accuracy, necessitating effective pre-processing denoising. Moreover, conventional AR extrapolation is essentially a scalar model and does not explicitly exploit the joint structure of the real and imaginary parts of the spectrum, which limits its robustness when dealing with narrow-band and low-SNR EMAT signals. These drawbacks motivate the introduction of a more robust vector Autoregressive framework together with an adaptive denoising front-end.

Variational Mode Decomposition (VMD) is a typical denoising method that decomposes signals into Intrinsic Mode Functions (IMFs) with specific center frequencies and limited bandwidth through a variational optimization framework. However, its effectiveness heavily depends on parameter selection. To address this limitation, researchers have developed various optimization strategies. Yin et al. [16] implemented a Genetic Algorithm (GA) for parameter optimization, though with limited computational efficiency; Hu et al. [17] utilized a Sparrow Search Algorithm (SSA) to achieve effective defect extraction under strong noise interference. Li et al. [18] applied a Marine Predators Algorithm (MPA) to effectively suppress background noise in transient electromagnetic signals. Additionally, Singular Value Decomposition (SVD) serves as an effective auxiliary denoising tool for random noise reduction. Current research demonstrates that combined VMD-SVD approaches have achieved satisfactory denoising results across multiple domains, including power load forecasting [19] and bridge monitoring [20].

This work introduces a Bayesian Vector Autoregression (BVAR) model into the spectral extrapolation framework for EMAT signals to overcome the aforementioned limitations. The method enhances extrapolation robustness and stability by incorporating prior distributions and multi-dimensional feature representations based on conventional autoregression. Building on this, we further propose an Adaptive Denoising and Bayesian Vector Autoregressive (AD-BVAR) spectral extrapolation method, which effectively mitigates the impact of noise on accuracy. The proposed methodology comprises three key stages. First, a Particle Swarm Optimization (PSO) algorithm is employed to adaptively optimize the parameters of VMD, ensuring an optimal match between the center frequencies and bandwidths of the decomposed modes. This step facilitates effective noise reduction and mode separation. Subsequently, SVD is applied to further eliminate random noise components, yielding intrinsic mode signals with high SNR. Finally, BVAR-based spectral extrapolation is performed on the denoised signal, which leverages Bayesian prior constraints and multi-dimensional feature coupling to achieve robust spectral extrapolation and high-resolution reconstruction.

## 2. Materials and Methods

### 2.1. PSO-VMD-SVD Adaptive Denoising Method

The core of VMD is to decompose a signal into Intrinsic Mode Functions (IMFs), each with a specific center frequency. Each mode is expressed as:

(1) $$x\left(t\right)=\sum_{n=1}^{N}\alpha_{n}h_{n}\left(t\right)$$

where $x\left(t\right)$ represents the signal, $h_{n}\left(t\right)$ denotes the IMFs, $\alpha_{n}$ is the amplitude of each mode, and $f_{n}$ is the center frequency of the respective IMF. The Dirac delta function is used to enforce the frequency constraints of each mode.

To solve for the parameters $\alpha_{n}$ and $f_{n}$ in the above equation, a penalty factor α and Lagrangian multiplier $\lambda_{n}$ are introduced, leading to an unconstrained minimization problem. The augmented Lagrangian function is formulated as:

(2) $$L(\alpha_{n},f_{n})=\sum_{n=1}^{N}\left[\|{\hat{u}}_{n}(t)-\alpha_{n}\cdot h_{n}(t){\|}^{2}+\lambda_{n}\left(L_{n}\right)\right]$$

The Alternating Direction Method of Multipliers (ADMM) is employed to iteratively update each component. The parameters $\alpha_{n}$, $f_{n}$, and $\lambda_{n}$ are alternately updated until convergence criteria are satisfied. After this decomposition process, the IMFs components are obtained. Effective components are selected using the correlation coefficient-envelope entropy criterion by calculating the correlation between each component and the original signal:

(3) $$\rho =\frac{\sum_{i=1}^{n}\left(x_{i}\cdot y_{i}\right)}{\sqrt{\sum_{i=1}^{n}x_{i}^{2}\sum_{i=1}^{n}y_{i}^{2}}}$$

This work employs the PSO algorithm for adaptive parameter searching, as detailed in Algorithm 1. Each particle represents a candidate combination of VMD parameters:

(4) $${\vec{p}}_{i}=(\alpha_{1},f_{1},\ldots ,\alpha_{n},f_{n})$$

**Algorithm 1:** PSO-VMD-SVD Adaptive Denoising AlgorithmInput: Raw detection signal x(t), particle swarm size $N_{P}$, maximum iteration count $Iter_{\max}$, number of modes k, penalty factor αStep 1: Randomly initialize particle positions k and velocities α;
Step 2: Repeat
     For i = 1: $N_{P}$ to do     Perform VMD on signal x(t)     Calculate permutation entropy of each IMF and screen useful components     Execute SVD denoising on noise-dominant IMFs     Reconstruct signal $y_{i}(t)$ and compute the personal best and global best
    End
     Update particle positions k and α, and subsequently calculate both the personal best and global best positions;
Step 3: Until maximum iteration count $Iter_{\max}$ is reached or convergence criteria are satisfied;
Step 4: Perform final VMD-SVD using optimal parameters and output denoised signal Y(t).

The particles iteratively approach the optimal parameter combination by dynamically updating their velocities and positions according to the following equations:

(5) $$v_{i+1}=\omega v_{i}+c_{1}r_{1}(p_{\mathrm{best}}-p_{i})+c_{2}r_{2}(g_{\mathrm{best}}-p_{i})$$

where $p_{i}$ denotes the position of the particle in the dimension, $v_{i}$ is its corresponding velocity, and ω is the inertia weight; $c_{1}$ and $c_{2}$ are the cognitive and social acceleration coefficients, $r_{1}$ and $r_{2}$ are random numbers uniformly distributed in [0,1], and $p_{best}$ and $g_{best}$ represent the particle’s historical best position and the global best position, respectively.

The fitness function is constructed based on the correlation coefficient–envelope entropy criterion:

(6) Fitness=max(ρ)−min(H)

where ρ represents the correlation coefficient between the denoised reconstructed signal and the original signal, and H denotes the envelope entropy. The PSO algorithm iteratively updates the parameter combinations to maximize the correlation and minimize the entropy, thereby obtaining the optimal VMD parameters.

In this fitness function, the correlation coefficient term evaluates the similarity be-tween the denoised signal and the original signal, encouraging the preservation of useful echo information and waveform morphology. At the same time, the envelope entropy term measures the concentration of energy in the denoised signal: a lower entropy indicates that the energy is more focused around the back-wall echoes and less spread over background noise. After obtaining the optimal VMD parameters through PSO, each effective mode component is denoised using SVD. For a given mode signal, the Hankel matrix H is constructed:

(7) $$H=\left[\begin{array}{cccc} x_{1} & x_{2} & \cdots & x_{n}\\ \;x_{2} & x_{3} & \cdots & x_{n+1}\\ \vdots & \vdots & \ddots & \vdots \\ \;x_{m} & x_{m+1} & \cdots & x_{m+n-1} \end{array}\right]$$

In the above construction, the one-dimensional mode signal is embedded into a Hankel matrix by arranging time-shifted copies of the signal as columns, so that neigh-boring columns share highly overlapping samples. For an ideal damped echo with strong temporal correlation, the resulting Hankel matrix is approximately low-rank, whereas random noise is spread across all singular components. The matrix is then decomposed using SVD:

(8) $$H=\;U\;\Sigma \;V^{T}$$

where U and V are orthogonal matrices, and Σ contains the singular values in descending order.

By applying a singular value threshold or retaining the largest singular values k, the noise components are effectively suppressed, and the denoised Hankel matrix $\tilde{H}$ is reconstructed as:

(9) $$\tilde{H}=\sum_{i=1}^{k}\sigma_{i}u_{i}v_{i}^{T}$$

This process enhances the SNR while preserving the main features of the effective VMD components. In particular, the algorithm now clearly states that each particle encodes a candidate pair of VMD parameters (the number of modes k and the penalty factor α). For a given particle, the corresponding k and α are used to perform VMD on the input EMAT signal. The particle swarm then updates these parameter pairs according to the personal-best and global-best fitness values, and the combination that yields the optimal fitness is finally selected as the VMD configuration.

### 2.2. Bayesian Vector Autoregression Model

Following signal denoising, precise thickness analysis of the echo signal is performed. Let the original A-scan signal collected by the electromagnetic probe in pulse-echo mode be x(t), and the denoised signal be Y(t), where t represents time. To enhance the spectral resolution of the A-scan signal, the frequency domain window corresponding to a drop of ε dB from the maximum amplitude of the signal spectrum Y(ω) is defined as the effective frequency band, with a frequency range from $\omega_{1}$ to $\omega_{2}$. Within the high SNR region (where ε ranges from 6 dB to 10 dB), effective frequency bands $\varepsilon_{l}(1\leq l\leq n)$ are selected to preserve sufficient spectral characteristics while reducing interference from low-SNR components.

In the traditional AR spectral extrapolation method, for each effective frequency band, the AR model parameters $a_{p}(1\leq p\leq k)$ are calculated using the Burg method. Forward and backward prediction formulas are employed to extrapolate the high-frequency and low-frequency components beyond the effective band:

(10) $${\hat{Y}}_{ij}\left(\omega \right)=-\sum_{p=1}^{k}a_{p}Y(\omega -p\omega_{s}/M)(\omega >\omega_{1})$$

(11) $${\hat{Y}}_{ij}\left(\omega \right)=-\sum_{p=1}^{k}a_{p}^{*}Y(\omega +p\omega_{s}/M)(0\leq \omega <\omega_{1})$$

where M is the number of sampling points at the sampling frequency $\omega_{s}$, $a_{p}$ represents the complex conjugate of $a_{p}^{*}$, and ${\hat{Y}}_{ij}\left(\omega \right)$ is the extrapolated spectrum, which can be transformed back into the time-domain signal $\hat{x}(t)$ through the inverse Fourier transform.

To further enhance the robustness and feature representation capability of spectrum modeling, a BVAR model is introduced to perform spectral extrapolation based on the traditional AR approach. The complex spectrum is within the effective frequency.

(12) $$Y_{t}=\mathbb{R}e\{\{Y_{t}\}\},Se\{\{Y_{t}\}\},t=1,\ldots ,T$$

Let the order of the VAR model be p. The model can be expressed as:

(13) $$Y_{t}=\sum_{i=1}^{p}A_{i}Y_{t-i}+u_{t}\sim N(0,\Sigma )$$

where $A_{i}$ is a 2 × 2 coefficient matrix, and Σ denotes the covariance matrix of the noise term. The parameters are estimated under a conjugate Normal–Inverse Wishart prior:

(14) $$vec\left(A\right)\sim N\;(Q_{0},\Sigma_{0}\;),\Sigma \sim DW\left(S_{0},\nu_{0}\right)$$

Here, $\nu_{0}$ represents the prior degree of freedom. The closed-form solutions for the coefficient matrix and covariance are given as:

(15) $$\hat{A}=\Omega (\Omega_{0}^{-1}vec(A_{0})+X^{T}\mathrm{vec}(Y)),\Omega ={\left(\Omega_{0}^{-1}+X^{T}X\right)}^{-1}X$$

where *X* denotes the lagged observation matrix. The extrapolation of the missing high and low frequency components is then recursively estimated as:

(16) $$\hat{Y}(\omega )=\sum_{i=1}^{p}{\hat{A}}_{i}\hat{Y}(\omega -i\Delta \omega )$$

The extrapolated complex spectrum satisfies conjugate symmetry, and the corresponding time-domain signal can be reconstructed through the inverse Fourier transform.

### 2.3. AD-BVAR Spectral Extrapolation Method

**Figure 1:**
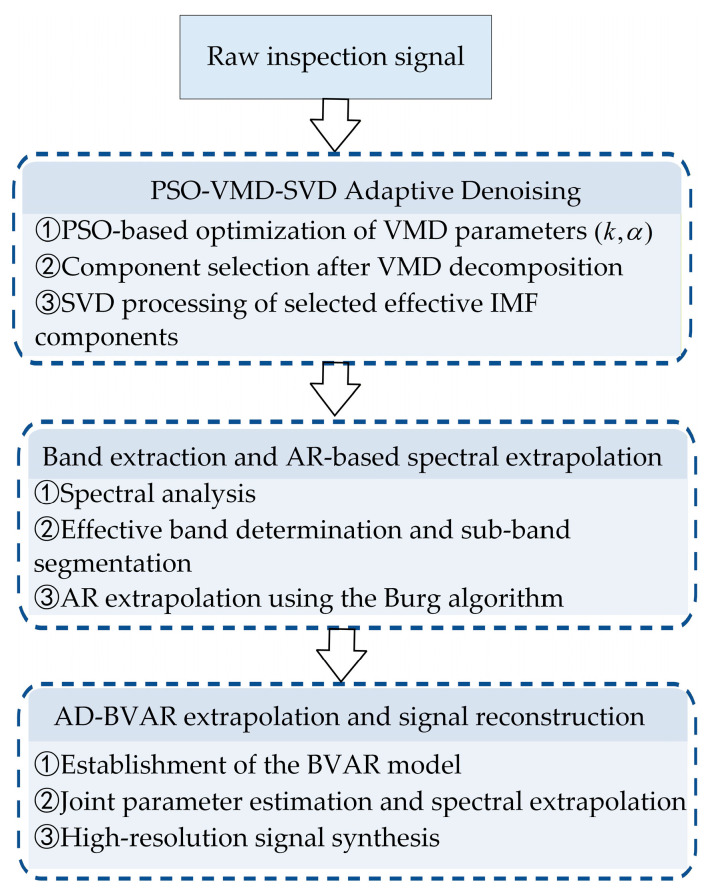
Flowchart of the AD-BVAR spectral extrapolation method.

To address the issues of limited spectral bandwidth, low SNR, and insufficient resolution in EMAT signals, this work proposes an AD-BVAR method. The overall framework, as illustrated in Figure 1, consists of three main stages: First, VMD is applied to the raw inspection signal to achieve multiscale decomposition, yielding several IMFs with distinct center frequencies. Effective components are automatically selected using a correlation coefficient–envelope entropy criterion, while residual noisy modes are further denoised in the energy domain using SVD. To eliminate uncertainties caused by manual parameter tuning, the PSO algorithm is introduced to globally optimize the key VMD parameters mode number k and penalty factor α, thereby achieving adaptive denoising and feature enhancement.

## 3. Simulation

The effectiveness of the proposed AD-BVAR spectral extrapolation method for EMAT thickness measurement was verified via numerical simulations performed in MATLAB R2025b. The simulation adopted typical steel acoustic parameters and was conducted on a workstation equipped with an Intel Core i7-10700K processor and 32 GB of RAM. The test block thickness was set to 12 mm, with a sampling rate of 200 MHz and 2000 sampling points. The excitation signal was modeled as a Gaussian-modulated pulse to simulate the ultrasonic echo generated by the EMAT, expressed as:

(17) $$s(t)=e^{-\frac{{\left(t-r\right)}^{2}}{2\sigma^{2}}}\cdot \sin(2\pi f_{0}t)$$

where $f_{0}$ is the probe center frequency (set to 5 MHz), t=0.5 μs represents the arrival time of the first echo, and σ is a pulse width control parameter governing the time domain spread and spectral bandwidth of the excitation.

The fundamental principle of ultrasonic A-scan thickness measurement is based on the ultrasonic echo method. When a longitudinal ultrasonic wave propagates through a specimen and encounters the bottom surface, a reflected echo is generated. The time interval Δt between the first echo and the bottom echo relates to the material thickness h and the wave propagation velocity *c* in the material through the following equation:

(18) $$d=\frac{c\cdot \Delta t}{2}$$

**Figure 2:**
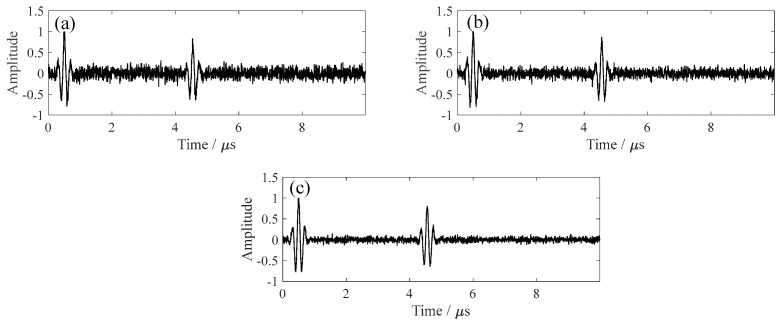
Simulated A−scan signals under different SNR levels: (**a**) 2 dB, (**b**) 5 dB, and (**c**) 10 dB.

To simulate electromagnetic and structural noise interference in real inspection environments, additive white Gaussian noise was added to the signal. The SNR was defined as the ratio of signal power to noise power. As shown in Figure 2, three typical SNR levels (2 dB, 5 dB, and 10 dB) were selected to represent strong, moderate, and weak noise conditions, forming a gradient of testing environments for robustness evaluation.

**Figure 3:**
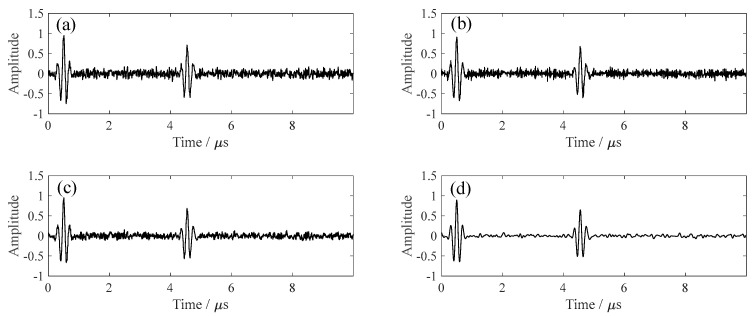
Denoising performance at SNR = 2 dB: (**a**) FIR, (**b**) Wavelet, (**c**) Fixed-parameter, and (**d**) PSO−VMD−SVD.

Four denoising algorithms were compared: (1) FIR band-pass filtering (2–40 MHz); (2) wavelet denoising; (3) fixed-parameter VMD-SVD (k = 3, σ = 1000); and (4) the proposed PSO-optimized adaptive denoising (PSO-VMD-SVD). As shown in Figure 3, under severe noise conditions (SNR = 2 dB), all algorithms partially restored the main echo, but PSO-VMD-SVD achieved the best performance with smoother baselines and superior noise suppression.

**Figure 4:**
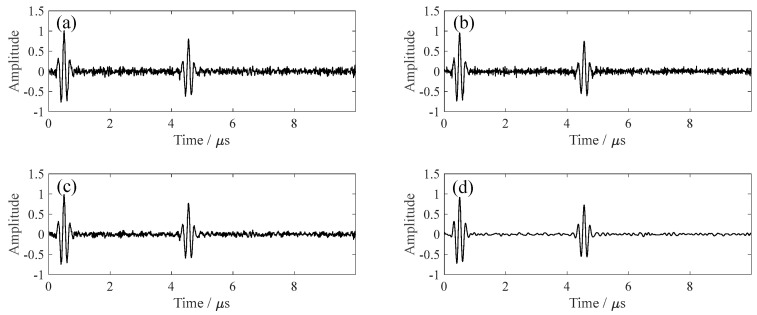
Denoising performance at SNR = 5 dB: (**a**) FIR, (**b**) Wavelet, (**c**) Fixed-parameter, and (**d**) PSO−VMD−SVD.

At SNR = 5 dB, the signal clarity improves significantly (Figure 4), and PSO-VMD-SVD demonstrates both effective background noise reduction and waveform preservation.

**Figure 5:**
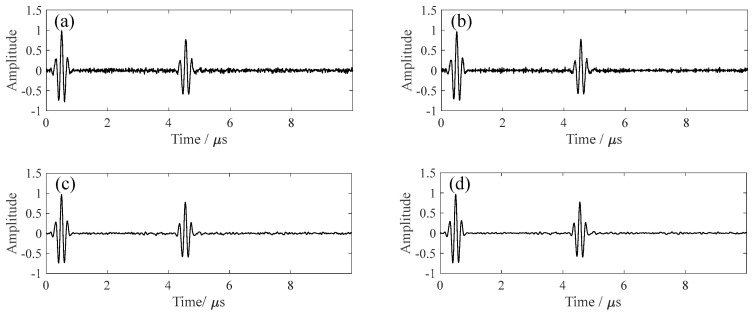
Denoising performance at SNR = 10 dB: (**a**) FIR, (**b**) Wavelet, (**c**) Fixed-parameter, and (**d**) PSO−VMD−SVD.

When SNR = 10 dB (Figure 5), all methods achieve satisfactory signal reconstruction, while PSO-VMD-SVD remains superior in waveform fidelity and baseline stability.

**Figure 6:**
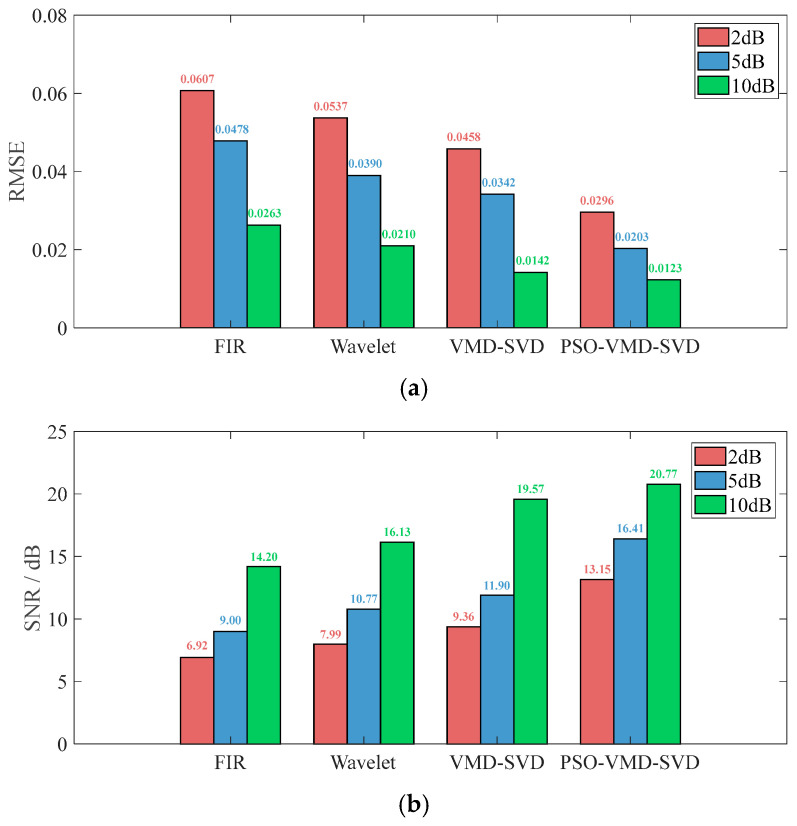
Comparison of denoising algorithms: (**a**) MSER and (**b**) SNR.

Figure 6 compares the denoising performance across SNR levels of 2, 5, and 10 dB. Figure 6a presents the Root Mean Square Error (RMSE), while Figure 6b shows the SNR. The results indicate that RMSE decreases with increasing SNR, and PSO-VMD-SVD consistently achieves the lowest RMSE and highest SNR gain. For instance, at an input SNR of 2 dB, PSO-VMD-SVD improves SNR by approximately 4 dB compared to FIR and by 1.2 dB over fixed-parameter VMD-SVD, confirming its superior capability in adaptive parameter optimization and robust denoising performance.

**Figure 7:**
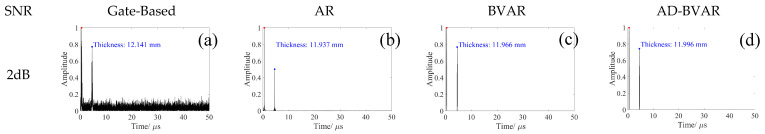

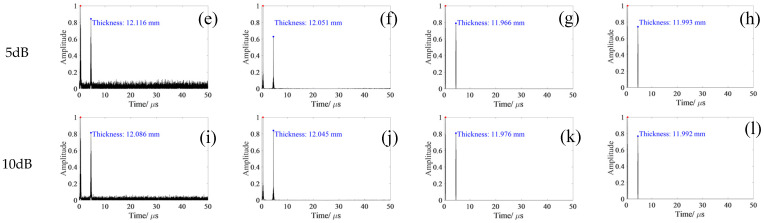
Thickness measurement results of different algorithms for a 12 mm steel specimen under three SNR levels. Subplots (**a**–**d**), (**e**–**h**), and (**i**–**l**) correspond to SNR = 2 dB, 5 dB, and 10 dB, respectively. For each SNR, (**a**,**e**,**i**) show the Gate-Based method, (**b**,**f**,**j**) the AR method, (**c**,**g**,**k**) the BVAR method, and (**d**,**h**,**l**) the proposed AD-BVAR method.

Figure 7 compares the thickness measurement results obtained by the Gate-Based, AR, BVAR, and proposed AD-BVAR methods under three SNR conditions (2 dB, 5 dB, and 10 dB). Figure 7a–d, Figure 7e–h, and Figure 7i–l correspond to input SNRs of 2 dB, 5 dB, and 10 dB, respectively; within each group, (a,e,i) show the Gate-Based method, (b,f,j) the AR method, (c,g,k) the BVAR method, and (d,h,l) the AD-BVAR method. The proposed PSO-VMD-SVD method exhibits superior noise suppression and waveform preservation across all conditions. The PSO-VMD-SVD method consistently outperforms the other methods across all SNR levels. At lower SNR (2 dB), noise suppression is critical, and the PSO-VMD-SVD method excels in reducing noise while maintaining the waveform integrity. As the SNR increases to 5 dB and 10 dB, the methods overall show improved performance, but the PSO-VMD-SVD method continues to demonstrate superior noise suppression and more accurate waveform retention compared to the Gate-Based, AR, BVAR, and AD-BVAR methods.

To quantify evaluate the thickness measurement performance of the various algorithms presented in Figure 7, the image data from each Figure 7a–l corresponding to the different SNRs were extracted and converted into Table 1.

**Figure 8:**
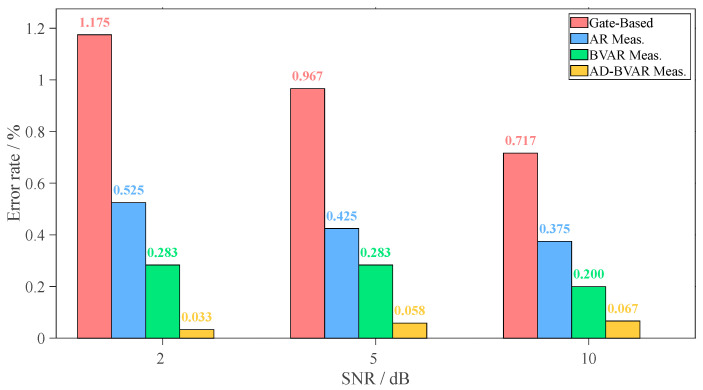
Error rates of different thickness measurement methods under various SNRs.

Figure 8 Error rates of different thickness measurement methods under various SNRs (Gate-Based, AR, BVAR, AD-BVAR) at three SNR levels (2 dB, 5 dB, and 10 dB). At 2 dB SNR, the AD-BVAR method achieves the lowest error (0.033%), far outperforming the Gate-Based method (1.175%). At 5 dB SNR, AD-BVAR still leads with 0.058%, followed by BVAR (0.283%), AR (0.425%), and Gate-Based (0.967%). At 10 dB SNR, AD-BVAR remains the best (0.067%), while all methods show improved error rates compared to lower SNR. Overall, the AD-BVAR method consistently exhibits superior performance across all SNR levels, with significant error reduction from 2 dB to 10 dB.

In practical EMAT measurements, the residual thickness error is mainly caused by limited SNR, imperfect suppression of background noise in the signal processing chain (e.g., time-of-flight picking and spectral extrapolation), and sensor-related factors such as lift-off variation and slight misalignment. The proposed AD-BVAR scheme mainly reduc-es the first two contributions by enhancing the SNR, sharpening the back-wall echoes and improving echo localization.

## 4. Experiment

### 4.1. Experimental Setup

**Figure 9:**
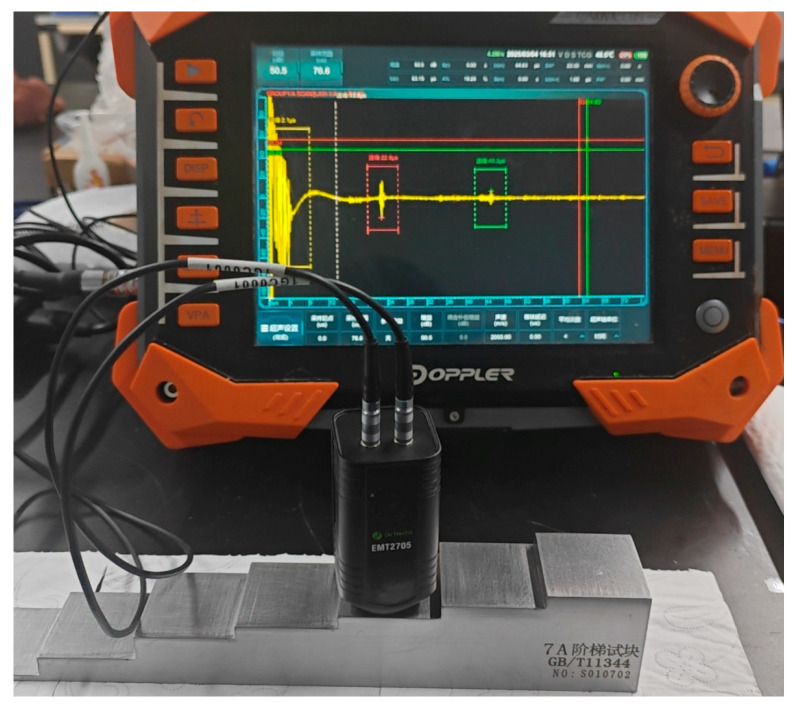
Test block and data acquisition setup.

The experiments utilized a NOVASCAN portable 32/128 phased array system from Guangzhou Doppler Electronic Technology Co., Ltd., Guangzhou, China with an EMAT probe center frequency of 4 MHz and a sampling frequency of 200 MHz. The wave propagation velocity in the steel medium was 5924 m/s. A seven-step test block with thicknesses of 3 mm, 12.5 mm, 24 mm, and 30 mm was used for measurement. All experiments were carried out at room temperature on carbon-steel step blocks, corresponding to typical ambient conditions after pipelines or components have cooled down in service, see Figure 9.

### 4.2. Experimental Results

**Figure 10:**
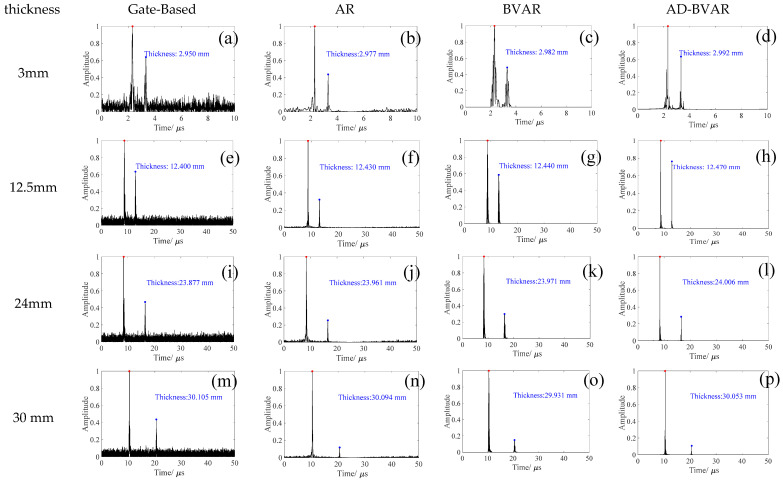
Comparison of signal extrapolation waveforms by different methods at various step thicknesses: (**a**–**d**) 3 mm, (**e**–**h**) 12.5 mm, (**i**–**l**) 24 mm, and (**m**–**p**) 30 mm.

To further validate the universality and accuracy stability of the AD-BVAR method across specimens of varying thicknesses, step blocks with thicknesses of 3 mm, 12.5 mm, 24 mm, and 30 mm were selected for this study. Their A-scan signals were extrapolated and used for thickness calculations, with the results shown in Figure 10.

For the 3 mm specimen, shown in Figure 10a–d, the AD-BVAR method (Figure 10d) measures 2.992 mm, with an error of just 0.008 mm, making it the most accurate. In comparison, the Gate-Based method (Figure 10a) shows the largest error of 0.050 mm. Similarly, for the 12.5 mm specimen in Figure 10e–h, the AD-BVAR method (Figure 10h) again provides the closest measurement of 12.470 mm, with a minimal error of 0.030 mm while the Gate-Based method (Figure 10e) has a larger error of 0.100 mm. Moving on to the 24 mm specimen, shown in Figure 10i–l, the AD-BVAR method (Figure 10l) measures 24.006 mm, with an error of only 0.006 mm outperforming the other methods. The Gate-Based method has the largest error of 0.123 mm. Finally, for the 30 mm specimen in Figure 10m–p, the AD-BVAR method (Figure 10p) measures 30.053 mm, with an error of 0.053 mm, maintaining its high accuracy. The BVAR method (Figure 10m) shows a larger error of 0.069 mm.

To quantitatively evaluate the thickness measurement accuracy of the different methods presented in Figure 10, the image data from each Figure 10a–p corresponding to the various specimen thicknesses were extracted and summarized in Table 2. All data points represent the mean from five independent repeated experiments to effectively mitigate random errors and provide a comprehensive comparison of the methods’ performance across the different thicknesses.

**Figure 11:**
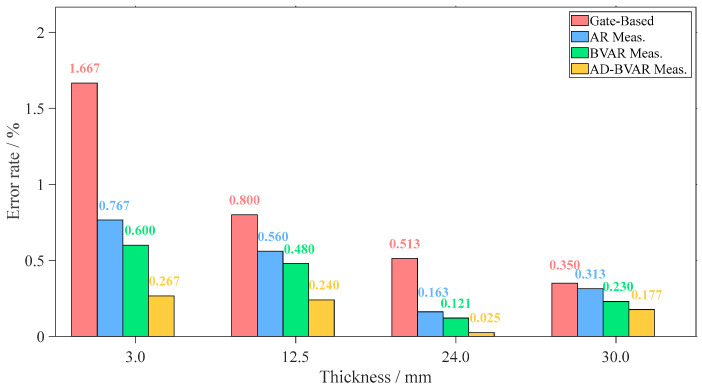
Error analysis under different thicknesses.

As shown on Figure 11, the AD-BVAR method consistently delivers the lowest error rates across all specimen thicknesses (3 mm, 12.5 mm, 24 mm, and 30 mm). For the 3 mm specimen, AD-BVAR achieves an error rate of 0.267%, significantly outperforming the Gate-Based method (1.667%). In the 12.5 mm sample, AD-BVAR maintains the lowest error at 0.240%, while the Gate-Based method remains the highest at 0.800%. For the 24 mm specimen, AD-BVAR excels with an error of just 0.025%, compared to 0.513% for the Gate-Based method. Finally, for the 30 mm specimen, AD-BVAR continues to lead with a 0.177% error, while the Gate-Based method shows the highest error of 0.350%.

In summary, the AD-BVAR method outperforms all other methods in terms of accuracy, consistently achieving error rates below 0.3%.

## 5. Conclusions

This paper proposes a high-precision electromagnetic ultrasonic thickness measurement method based on Adaptive Denoising and Bayesian Vector Autoregressive Spectral Extrapolation (AD-BVAR). The proposed method effectively addresses the challenges associated with limited frequency bandwidth and low SNR in EMAT signals, which are key factors contributing to insufficient thickness measurement accuracy. The primary research findings are summarized as follows:

1. An adaptive denoising method was developed, referred to as PSO-VMD-SVD. This method utilizes the PSO algorithm to automatically determine the key parameters for VMD, combined with SVD for joint denoising. Experiments demonstrated that this method improves the SNR by approximately 6 dB compared to conventional FIR filtering at an input SNR of 2 dB, effectively suppressing noise while preserving crucial waveform details.

2. A BVAR model for spectral extrapolation was established. By incorporating Bayesian priors, this model performs robust joint modeling and extrapolation of the real and imaginary parts of the spectrum, overcoming the noise sensitivity inherent in the traditional AR method and significantly enhancing the quality of full-band spectrum reconstruction for A-scan signals.

3. Both numerical simulations and experimental validations were performed to assess the effectiveness of the proposed method under varying noise levels and sample thicknesses. At SNR levels of 2 dB, 5 dB, and 10 dB, the proposed AD-BVAR approach consistently yields thickness estimations that are closest to the reference values. Furthermore, for specimens with different thicknesses of 3 mm, 12.5 mm, 24 mm, and 30 mm, the AD-BVAR method achieves the highest measurement accuracy and stability among all compared techniques.

The main limitation of the proposed AD-BVAR scheme is the additional computational cost introduced by the PSO-VMD-SVD front-end, which is acceptable for the single channel EMAT A-scan signals studied here, but may become burdensome in high-throughput or imaging applications, where future work will focus on algorithm acceleration.

In summary, the developed AD-BVAR method, integrating intelligent optimization, adaptive signal decomposition, and Bayesian statistical inference, provides a robust and reliable technical pathway for high-precision EMAT thickness measurement in low-SNR environments. Future work will focus on incorporating deep learning and curved surface scattering models to further enhance the intelligent inspection and evaluation capabilities for complex components.

## Figures and Tables

**Table 1:** Thickness measurement results of different algorithms under different SNR levels.

SNR (dB)	Gate-Based (mm)	AR Meas. (mm)	BVAR Meas. (mm)	AD-BVAR Meas. (mm)
2.000	12.141	11.937	11.966	11.996
5.000	12.116	12.051	11.966	11.993
10.000	12.086	12.045	11.976	11.992

**Table 2:** Performance comparison of different thickness measurement methods.

Actual Thickness (mm)	Gate-Based (mm)	AR Meas. (mm)	BVAR Meas. (mm)	AD-BVAR Meas. (mm)
3.000	2.950	2.977	2.982	2.992
12.500	12.400	12.430	12.440	12.470
24.000	23.877	23.961	23.971	24.006
30.000	30.105	30.094	29.931	30.053

## Data Availability

The raw data supporting the conclusions of this article will be madeavailable by the authors on request.

## Abbreviations

The following abbreviations are used in this manuscript:

AD-BVAR	Adaptive Denoising and Bayesian Vector Autoregressive
AR	Autoregressive
BVAR	Bayesian Vector Autoregressive
EMAT	Electromagnetic Acoustic Transducer
FIR	Finite Impulse Response
GA	Genetic Algorithm
IMFs	Intrinsic Mode Functions
MPA	Marine Predators Algorithm
PSO	Particle Swarm Optimization
RMSE	Root Mean Square Error
SNR	Signal-to-Noise Ratio
SSA	Sparrow Search Algorithm
SVD	Singular Value Decomposition
VMD	Variational Mode decomposition
